# Seasonal change and phylogenetic position of *Kamegainema cingula* (Nematoda: Dracunculidae) parasitic in Japanese giant salamanders

**DOI:** 10.1016/j.ijppaw.2025.101052

**Published:** 2025-02-28

**Authors:** Karin Tsuchida, Misako Urabe, Kanto Nishikawa

**Affiliations:** a Graduate School of Global Environmental Studies, Kyoto University, Yoshidahon-cho, Sakyo Ward, Kyoto City, Kyoto, 606-8501, Japan; b Faculty of Environmental Sciences, The University of Shiga Prefecture, 2500 Hassaka-cho, Hikone City, Shiga, 522-8533, Japan; c Graduate School of Human and Environmental Studies, Kyoto University, Yoshidahon-cho, Sakyo Ward, Kyoto City, Kyoto, 606-8501, Japan

**Keywords:** Amphibian, Asia, Ectoparasite, Helminth, Phylogeny

## Abstract

*Kamegainema cingula* (Linstow, 1902) (Nematoda: Dracunculidae) parasitizes subcutis of cryptobranchid salamanders in Japan and the U.S.A. *Kamegainema* is a monotypic genus including only *K. cingula*. Here, we analyzed the phylogenetic relationship of *K. cingula* in other dracunculid and micropleurid species. We also reported the seasonal change of the present species in the infection rate in the skin of *Andrias* species in Kyoto and Hyogo prefectures, Japan. We collected this species from the skin of *Andrias japonicus* and hybrids (*A. japonicus* × *Andrias davidianus*) from spring to early summer. Female *K. cingula* likely mature and release larvae during this season in Japan. In addition, *K. cingula* formed a sister clade to *Micropleura* as well as *Dracunculus* in our phylogenetic analysis.

## Highlights

• Seasonal change of *Kamegainema cingula* parasitic in the subcutis of salamanders of *Andrias*.
• Phylogenetic position of the genus *Kamegainema* in the family Dracunculidae.
• New information on phylogenetic relationship in Dracunculoidea.

## Graphical abstract

**Figure:**
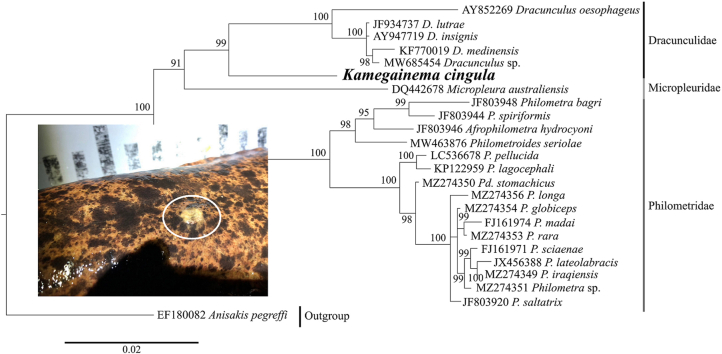

## 1 Introduction

*Kamegainema cingula* (Linstow, 1902) Hasegawa, Doi, Araki and Miyata, 2000 (Nematoda: Dracunculidae) is an endemic helminth to cryptobranchid salamanders. Linstow (1902) originally described this species as *Filaria cingula* recovered from Japanese giant salamanders, *Andrias japonicus* (Temminck, 1836) (Urodela: Cryptobranchidae), which were carried from Japan to the Netherlands and reared in the Hamburg Zoological Garden. Krecker (1915) reported *F. cingula* from the skin of the hellbender *Cryptobranchus alleganiensis* (Daudin, 1803), in the Ohio River, U.S.A.

Hasegawa et al. (2000) reported the present species from the skin of *A. japonicus* in Hyogo Prefecture and established a new genus *Kamegainema* Hasegawa, Doi, Araki and Miyata, 2000 based on this species. In addition, Hasegawa and Ikeda (2003) detected *K. cingula* in the abdominal cavity of *C. alleganiensis* in the Niangua River, U.S.A. Only female specimens have been recorded from both Japan and the U.S.A. In addition, all these records were concentrated during March and May as adults and indicated a possibility if there is a seasonal change in the female nematodes coming outside from the skin. Kumazawa (2016) reported that *Mesocyclops woutersi* van de Velde, 1987 (Copepoda: Cyclopidae) served as the first intermediate host and both cultured *Oryzias latipes* (Temminck and Schlegel, 1846) (Osteichthyes: Adrianichthyidae) and larval *Hynobius hirosei* Lantz, 1931 (Amphibia: Hynobiidae) could be infected with L3 larvae of *K. cingula* in the experimental infection; nevertheless, this nematode has not been recorded from other aquatic organisms excepting cryptobranchid salamanders under wild conditions.

The family Dracunculidae Stile, 1907 (Nematoda: Dracunculoidea) consists of four genera following Moravec (2004): *Dracunculus* Reichard, 1759, *Avioserpens* Wehr and Chitwood, 1934, *Protenema* Petter and Planelles, 1986, and *Kamegainema*. Hodda (2022) proposed that Dracunculidae consists of three genera (*Avioserpens*, *Dracunculus*, and *Lockenloia* Adamson and Caira, 1991) while *Kamegainema* and *Protenema* were transferred to Micropleuridae Baylis and Daubney, 1926 (Dracunculoidea) based on the only literature reviews. Here, we assumed the classification by Moravec (2004) because *Lockenloia* is a group of *incertae sedis* and no amendment of diagnosis in *Kamegainema* and *Protenema* in Hodda (2022). The genera *Dracunculus* and *Avioserpens* are composed of species using mammals or reptiles and birds, respectively. On the other hand, the genera *Kamegainema* and *Protenema* utilize amphibians as definitive hosts.

Here, we analyzed the phylogenetic position of *K. cingula* using the nuclear 18S ribosomal DNA (rDNA) and the mitochondrial cytochrome *c* oxides subunit I (*cox1*) gene. Additionally, we reported the seasonal change of its detection rate from the subcutis of *Andrias* in Kyoto and Hyogo prefectures, Japan, which helps to understand the life cycle of *K. cingula.*

## 2 Materials and methods

### 2.1 Collections of host and parasite

**Fig. 1:**
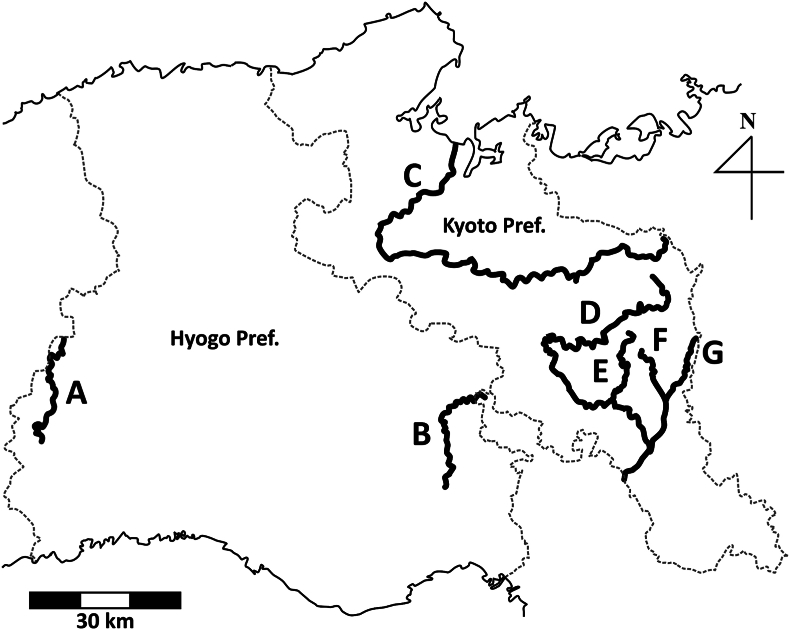
Map of the examined river in the survey for *Andrias japonicus* in Kyoto and Hyogo prefectures, Japan. A, Sayo River (Chikusa River Basin); B, Hatsuka River (Muko River Basin); C, Yura River (Yura River Basin); D, Katsura River (Yodo River Basin); E, Kiyotaki River (Yodo River Basin); F, Kamo River (Yodo River Basin); and G, Takano River (Yodo River Basin).

We collected ectoparasitic nematodes from the skin of *A. japonicus* and hybrids (*A. japonicus* × *A. davidianus*) from February to September 2023 in Kyoto Prefecture, Japan, within the survey for Japanese giant salamanders under the permission of the Japanese Cultural Affair (Table 1; Fig. 1). Specimens of nematodes collected in Hyogo Prefecture, Japan, were provided from the Nature Conservation Society of Hyogo Prefecture (Table 1; Fig. 1). The visual encounter surveys for Japanese giant salamanders involved 1–6 people walking slowly from downstream to upstream at night (from 19:00–22:00) while using flashlights and capturing all encountered salamanders with a dip-net. If there is a possibility of injuring *A. japonicus* by extracting the nematode body from their skin, we just counted nematodes found on the skin without extraction. The intensity and prevalence of *K. cingula* were calculated based on the number of female nematodes coming out from the host skin because we have no method to confirm the inside status of *A. japonicus*. The host species were genetically identified following Yoshikawa et al. (2012) and classified into *A. japonicus*, *A. davidianus*, and their hybrids. We avoid stating the distinct localities from the conservation perspective of the special natural monument. Examined hybrid salamanders were deposited in the herpetological collection of the Graduate School of Human and Environmental Studies, Kyoto University (KUHE). We classified collected female nematodes by maturity into three stages as follows: immature (without eggs and larvae), gravid with eggs (having eggs in the uterus), and gravid with larvae (having L1 larvae in the uterus). We fixed collected nematodes in 70–100% ethanol and morphologically identified specimens cleared in undiluted lactic acid solution under the microscope, following Hasegawa et al. (2000). We deposited the examined specimens in the Zoological Collection of Kyoto University (KUZ) (catalog nos. KUZ Z6015–Z6017).

**Table 1:**
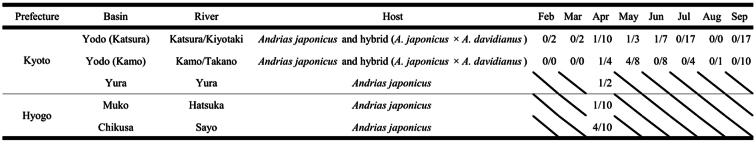
List of examined hosts in 2023. Diagonal line, no survey month; the number of infected individuals/total number of captured individuals.

### 2.2 Phylogenetic analysis

We cut out a piece of tissue from fixed specimens and extracted their genomic DNA by alkaline lysis method (Bimboim and Doly, 1979). Partial sequences of 18S rDNA and *cox1* were amplified through the polymerase chain reaction (PCR) using a 2720 thermal cycler (Applied Biosystems, Inc. Massachusetts, USA). PCR was performed in 20 μl PCR reaction mixture containing 10 μl of 2 × Gflex PCR Bufer (Takara Bio, Inc. Shiga, Japan), 7.1 μl of Milli-Q water, 0.4 μl of Tks Gflex DNA Polymerase (Takara Bio, Inc.), 1 μl of each primer in 10 μmol/L and 0.5 μl of each template, using the thermal cycler. The thermocycling process for 18S rDNA was as follows: 5 min at 94 °C, 35 cycles of 40 s at 94 °C, 40 s at 50 °C, 2 min at 68 °C, and a final extension for 10 min at 68 °C. For *cox1*, 1 min at 94 °C, 40 cycles of 10 s at 94 °C, 15 s at 45 °C, 1 min at 68 °C, and a final extension for 7 min at 68 °C. We used the primer set PhilonemaF (5′-gcctataatggtgaaaccgcgaac-3′) and PhilPCRr (5′-ccggttcaagccactgcgatta-3′) for 18S rDNA (Černotíková et al., 2011), and LCO1490 (5′-ggtcaacaaatcataaagatattgg-3′) and HCO2198 (5′-taaacttcagggtgaccaaaaaatca-3′) for *cox1* (Folmer et al., 1994). PCR products were separated on electrophoresis gels with 1 μl Midorigreen Direct (NIPPON Genetics Co., Ltd, Tokyo, Japan), refined using the Wizard® SV Gel and PCR Clean-up System (Promega, Wasinton, U.S.A.), and sequenced directly (DNA sequencing service of FASMAC Co., Ltd., Kanagawa, Japan). We deposited the obtained sequences in the International Nucleotide Sequence Database (INSD) via the GenBank (accession nos. 18S: PV123245; *cox1*: PV125758–PV125762).

We made the data set of the obtained sequences and sequences deposited in the NCBI Genbank based on the result of the BLAST search in MEGA 7 (Kumar et al., 2016) and aligned them using MAFFT 7.222 (Katoh and Standley, 2013) and trimAl (Capella-Gutierrez et al., 2009) (Table 2). Outgroups were selected from non-dracunculid species based on the similarity of sequence with the species analyzed in this study. Phylogenetic trees were constructed by the maximum likelihood (ML) method with Raxml-GUI (Edler et al., 2021). We selected the GTR + G + I (Tavaré, 1986) model with a default gamma parameter of 4 as the optimum substitution model for the ML phylogenetic analysis of both 18S rDNA and *cox1* using ModelTest-NG (Flouri et al., 2014; Darriba et al., 2020). The probabilities were tested by the bootstrap analysis of 1000 replicates. The phylogenetic trees were also constructed by the Bayesian Inference (BI) method with MrBayes v3.2.6 (Ronquist et al., 2012). We selected the K80 + G + I (Kimura, 1980) and GTR + G models for the BI analysis of the 18S and *cox1*, respectively, using Kakusan4 (Tanabe, 2011) based on Schwarz's Bayesian information criterion (Schwarz, 1978). The BI analysis was conducted in two independent runs of five million generations, each with four Markov chains, and sampled the resulting trees every 100 generations. The convergence was determined by Tracer 1.7 (Rambaut et al., 2018) and the initial 10 % of trees were discarded as burn-in. The constructed trees by both methods were visualized in FigTree v1.4.4.

**Table 2:** List of dracunculoid sequences used for analysis data set.

Species	Host		Source	18S eDNA	*cox1*
**Dracunculidae**					
***Kamegainema cingula***	*Andrias japonicus* (Sayo R.)	<Amphibia>	This study	identical to PV123245	PV125758
	*A. japonicus* (Hatsuka R.)	<Amphibia>	This study	identical to PV123245	PV125759
	*A. japonicus* (Kiyotaki R.)	<Amphibia>	This study	PV123245	PV125760
	*A. japonicus* (Yura R.)	<Amphibia>	This study	identical to PV123245	PV125761
	*A. japonicus × Andrias davidianus* (Kamo R.)	<Amphibia>	This study	–	PV125762
	*A. japonicus × A. davidianus* (Takano R.)	<Amphibia>	This study	–	identical to PV125762
*Dracunculus lutrae*	*Lutra lutra/Lontra canadensis*	<Mammalia>	Laetsch et al. (2012)/Elsasser et al. (2009)	JF934737	EU646597
*Dracunculus insignis*	*Procyon lotor/L. canadensis*	<Mammalia>	Bimi et al. (2005)/Yabsley et al. (2024)	AY947719	PP384424
*Dracunculus* sp.	*Homo sapiens*	<Mammalia>	Natalini et al. (2023)	MW685454	
*Dracunculus medinensis*	*Homo sapiens*	<Mammalia>	Eberhard et al. (2014)/Direct submittion	KF770019	HQ216219
*Dracunculus oesophageus*	*Natrix natrix*	<Reptilia>	Wijová et al. (2006)	AY852269	
*Dracunculus jaguape*	*Lontra longicaudis*	<Mammalia>	Natalini et al. (2023)		OR575050
**Micropleuridae**					
*Micropleura australiensis*	*Crocodylus johnsoni*	<Reptilia>	Wijová et al. (2006)	DQ442678	
**Philometridae**					
*Philometra bagri*	*Bagrus bajad*	< Actinopterygii>	Černotíková et al. (2011)	JF803948	
*Philometra spiriformis*	*Lates niloticus*	< Actinopterygii>	Černotíková et al. (2011)	JF803944	
*Philometra pellucida*	*Arothron nigro*	< Actinopterygii>	Iwaki et al. (2020)	LC536678	
*Philometroides seriolae*	*Seriola quinqueradiata*	< Actinopterygii>	Choe and Eom (2022)	MW463876	
*Afrophilometra hydrocyoni*	*Hydrocynus forskahlii*	< Actinopterygii>	Černotíková et al. (2011)	JF803946	
*Philometra longa*	*Belone belone*	< Actinopterygii>	Barton et al. (2022)	MZ274356	
*Philometra* sp.	*Lutjanus johnii*	< Actinopterygii>	Barton et al. (2022)	MZ274351	
*Philometra lagocephali*	*Lagocephalus lunaris*	< Actinopterygii>	Wang et al. (2015)	KP122959	
*Philometra sciaenae*	*Pennahia argentata*	< Actinopterygii>	Quiazon et al. (2014)	FJ161971	
*Philometra rara*	*Hyporthodus haifensis*	< Actinopterygii>	Barton et al. (2022)	MZ274353	
*Philometra iraqiensis*	*Mugil cephalus*	< Actinopterygii>	Barton et al. (2022)	MZ274349	
*Philometroides stomachicus*	*Protonibea diacanthus*	< Actinopterygii>	Barton et al. (2022)	MZ274350	
*Philometra saltatrix*	*Pomatomus saltatrix*	< Actinopterygii>	Černotíková et al. (2011)	JF803920	
*Philometra globiceps*	*Uranoscopus scaber*	< Actinopterygii>	Barton et al. (2022)	MZ274354	
*Philometra lateolabracis*	*Epinephelus costae*	< Actinopterygii>	Genc and Keskin (2012)	JX456388	
*Philometra madai*	*Pagrus major*	< Actinopterygii>	Quiazon et al. (2014)	FJ161974	
**Outgroup**					
*Anisakis pegreffi*	*Caretta caretta*	<Reptilia>	Nadler et al. (2007)	EF180082	
*Clavinema parasiluri*	*Silurus asotus*	< Actinopterygii>	Zou et al. (2022)		NC_070136

## 3 Results

### 3.1 Seasonal change of detection from skin

**Fig. 2:**
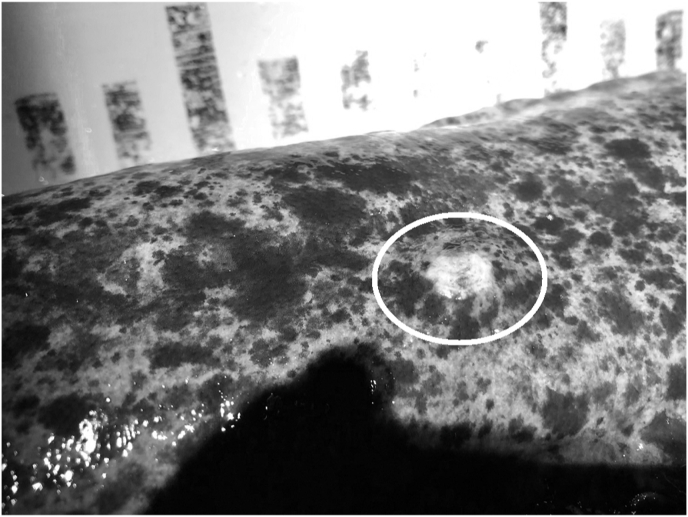
Photo of *Kamegainema cingula* in the skin of *Andrais japonicus*: white circle showing the infection site of the nematode.

*Kamegainema cingula* parasitized the dorsal and lateral (occasionally ventral) subcutaneous of hosts and often formed a cream-yellow spiral in the skin, sometimes getting out its partial body from the skin (Fig. 2). All of the collected specimens were females. We detected *K. cingula* from April to June (Table 1). Immature specimens were found only in April in this study (Table 3).

**Table 3:**
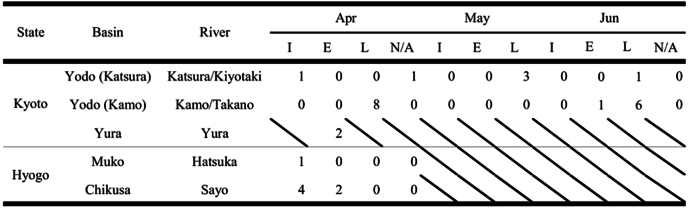
Examined *Kamegainema cingula* (I immature, E having eggs in uterus, L having L1 larvae in uterus, N/A no data).

We found three individuals infected with *K. cingula* (mean intensity: 1.7; prevalence: 5.1%) out of 58 of giant salamanders that were collected in all seasons and investigated in the Katsura River Basin (Kyoto Pref.). During April to June when there were salamanders infected, three of 20 individuals were infected (17%). While we found five individuals infected with *K. cingula* (mean intensity: 3.0; prevalence: 14.3%) out of 35 giant salamanders that were collected in the Kamo River Basin (Kyoto Pref.) during the examination. In April and May, five of 12 individuals were infected (41.7%). In the Yura River Basin, we found one infected individual (intensity: 2.0) out of two collected in April. In Hyogo Prefecture, we found one individual infected with *K.cingula* (intensity: 1.0; prevalence: 10%) out of 10 collected in April in the Hatsuka River. We found five individuals infected (mean intensity: 1.2; prevalence: 45.5%) out of 11 collected in April in the Sayo River.

### 3.2 Phylogenetic analysis

**Fig. 3:**
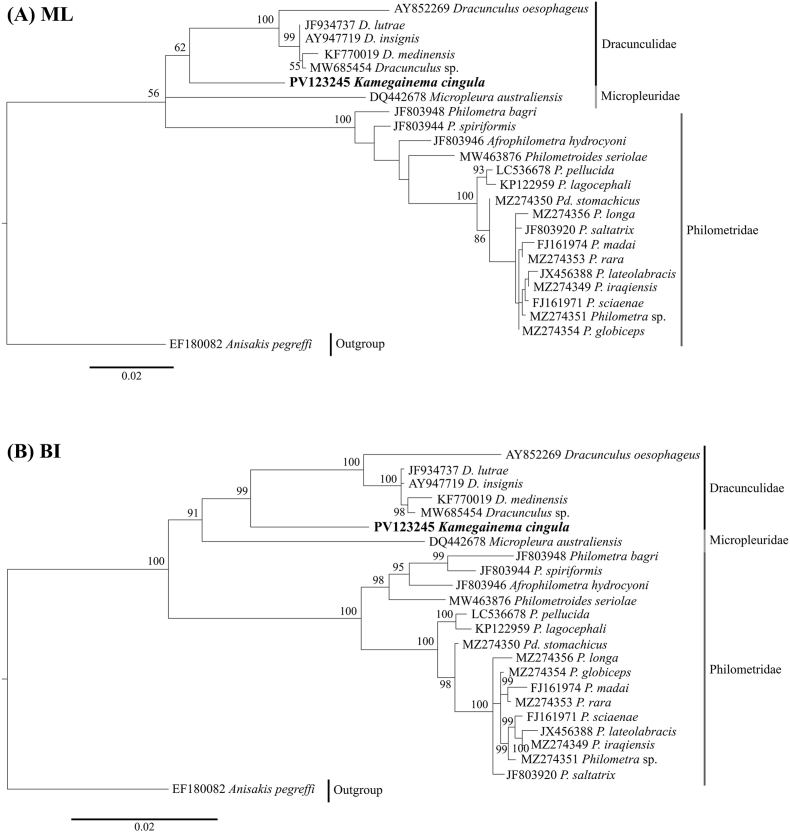
Phylogenetic tree of 18S rDNA sequences of dracunculoids. Bootstrap values and posterior probabilities were shown if they exceeded 50. (A) maximum likelihood method and (B) Bayesian inference method.

We successfully sequenced 1593 bp of 18S rDNA from three individuals of *K. cingula*, which was identical among all three specimens. We constructed phylogenetic trees with a total of 1574 bp of 18S rDNA region. The ML and BI trees differed in topology (Fig. 3). Neither the phylogenetic position of *K. cingula* nor *Micropleura australiensis* Moravec, 2004 (Micropleuridae) were resolved in the ML tree. In the BI tree, *K. cingula* first blanched off in dracunculids and formed a sister clade to *Dracunculus* species. Then *Dracunculus oesophageus*, which parasitize the snake, diverged from the clade of other *Dracunculus* species, all of which parasitize mammals (Table 2). Additionally, the representative of Micropleuridae (*M*. *australiensis*) occupied an intermediate position between dracunculid and philometrid species.

**Fig. 4:**
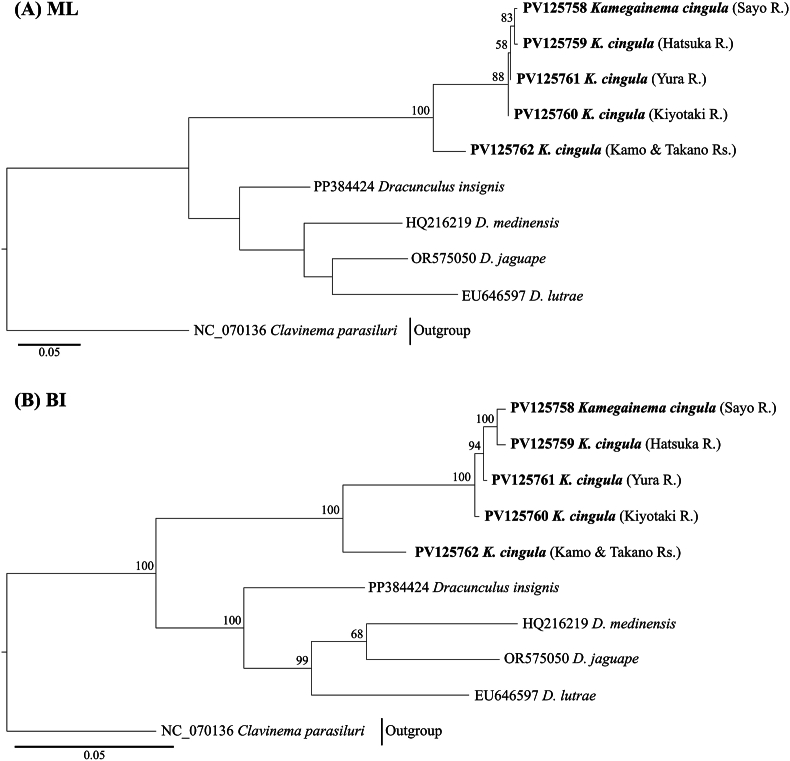
Phylogenetic tree of *cox1* sequences of dracunculids. Bootstrap values and posterior probabilities were shown if they exceeded 50. (A) maximum likelihood method and (B) Bayesian inference method.

We successfully sequenced 614 bp of *cox1* from six individuals of *K. cingula*, which included five haplotypes. Each haplotype differed by 0.2–6.4% as uncorrected *p*-distance (Table 4). We constructed phylogenetic trees with a total of 585 bp of *cox1* region. Haplotypes of *K. cingula* formed a well-supported clade and their relationship was approximately identical in both trees (Fig. 4). The *K. cingula* haplotype from the Kamo River Basin (Kamo and Takano rivers) first blanched off followed by that of Katsura River Basin (Kiyotaki River). The *K. cingula* haplotypes from rivers in Hyogo Prefecture (Sayo and Hatsuka rivers) formed a sister clade to those from the Yura River, Prefecture.

**Table 4:** Uncorrected *p*-distance of *cox1* fragments of *Kamegainema cingula*.

	Haplotype	1	2	3	4	5
1	PV125758 (Sayo)		0.003	0.007	0.005	0.064
2	PV125759 (Hatsuka)			0.007	0.005	0.064
3	PV125760 (Kiyotaki)				0.002	0.059
4	PV125761(Yura)					0.060
5	PV125762 (Kamo)

## 4 Discussion

*Kamegainema cingula* was detected from the skin of Japanese and hybrid giant salamanders from April to June, and not detected in February, March, and July to September, in Kyoto and Hyogo prefectures. All individuals collected from the skin were females, most of which were mature, suggesting that female *K. cingula* mature under the skin and release L1 larvae during spring and early summer in Japan. Because immature females were detected only in April, females may migrate from the deep part of the body to the subcutaneous and mature there during April. In the Missouri River Basin in the U.S.A., the gravid females of *K. cingula* have been reported from the hellbender in the spring (Krecker, 1915; Hasegawa et al., 2000). These facts agree with our suggestion that the present species release L1 larvae from the host body surface to the water during the spring.

The life history of Dracunculidae involves aquatic crustaceans, for instance, copepods, ostracods, and branchiurids, as intermediate hosts and vertebrates as definitive hosts (Moravec, 2004). One of the dracunculid species *Protenema longispicula* Petter and Planelles, 1986, which uses amphibians as definitive hosts, utilizes a species of the genus *Cyclops* (Copepoda) as intermediate hosts. They molt twice in copepods to become infective L3 larvae (Petter and Planelles, 1986). The definitive hosts are infected with the nematodes by ingesting intermediate hosts with L3 larvae.

Neither the L4 larvae of *K. cingula* nor adult males have been reported from the intestine of *Andrias* and *Cryptobranchus* (e.g. Hasegawa et al., 2002; Tsuchida et al., 2021). Considering these previous studies, the life cycle of *K. cingula* is expected as follows: its larvae develop within aquatic crustaceans from the L1 to L3 stages, and infect cryptobranchid salamanders orally by ingesting infected crustaceans. The L3 larvae grow up to be mature adults somewhere inside the body of salamanders and copulate there. After copulation, males would immediately die and females migrate toward the skin to release larvae.

Chabaud and Bain (1994) suggested that the superfamily Dracunculoidea Stile, 1907 might originate from the family Chitwoodchabaudiidae (Seuratoidea) which today involves the sole species, *Chitwoodchabaudia skryabini* Puylaert, 1970, parasitizing the frogs of the genus *Xenopus* in Africa (Tinsley, 1981). They also considered that the ancestral dracunculoid species could occur as the genus *Micropleura* Linstow, 1902 (Dracunculoidea: Micropleuridae) in the crocodiles and turtles in the Triassic or Jurassic, based on morphological characteristics. Micropleurid species have a similar polygenous lifecycle as dracunculids with small crustaceans like *Cyclops* and vertebrates (Siddiqi and Jairajpuri, 1963). Cleveland et al. (2018) suggested that reptiles would be original hosts of *Dracunculus* based on the higher species diversity of *Dracunculu*s in reptiles than that in mammals. Previous phylogenetic studies showed that Dracunculidae formed a sister group to Micropleuridae and Philometridae Baylis and Daubney, 1926 with an indefinite relationship (Černotíková et al., 2011). In addition, Hopkins et al. (2018) considered that frogs might be more susceptible to infecting mammalian *Dracunculus* and serve as more significant paratenic or transport hosts than fishes. Although there is no phylogenetic analysis of dracunculid species parasitizing amphibians in adulthood, it has been assumed that the primitive dracunculid species evolved in amphibians and switched their definitive hosts to reptiles and then, to mammals. Our phylogenetic analysis showed that Micropleuridae occupied the intermediate position between Dracunculidae and Philometridae. Moravec (2004) suggested that most dracunculoid species are found in aquatic environments because their life history involves aquatic crustaceans as intermediate hosts and the transmission to vertebrate hosts occurs in aquatic environments. This idea permits us to consider that the life history of dracunculoids would have evolved from the autogenic lifecycle in aquatic environments. Both Philometridae and Micropleuridae are composed of autogenic species within aquatic environments. On the other hand, Dracunculidae involves both autogenic and allogenic species: *Kamegainema* and *Protenema* are autogenic groups within freshwater environments, while *Dracunculus* and *Avioserpens* are allogenic groups including freshwater and terrestrial environments. Thus, Dracunculidae would be one of the important taxa in this superfamily regarding the shift of life history. Further study is required to reveal the phylogenetic relationship of dracunculid species.

## 5 Conclusions

We found that female *Kamegainema cingula* become mature under the skin of *Andrias* and release larvae into the water during the spring-early summer period (April to June) in western Japan. The present species formed a sister clade to *Dracunculus*. In the dracunculoidean cluster, Micropreulidae occupied the intermediate position between Philometridae and Dracunculidae.

## Database

INSD (accession nos. PV123245, PV125758–PV125762).

## CRediT authorship contribution statement

**Karin Tsuchida:** Writing – original draft, Visualization, Methodology, Investigation, Formal analysis, Data curation, Conceptualization. **Misako Urabe:** Writing – review & editing, Supervision. **Kanto Nishikawa:** Writing – review & editing, Resources.

## Funding

This research was generously supported by philanthropic gifts from Y. Arai, K. Haruki, M. Imai, K. Nishikawa, A. Kunihiro, K. Okamoto, T. Watahiki, K. Yamashita, GS Craft Co. Ltd, and 513 supporters through the Readyfor crowdfunding.

## Declarations of interest

None.
